# Evidence of rapid rise in population immunity from SARS-CoV-2 subclinical infections through pre-vaccination serial serosurveys in Pakistan

**DOI:** 10.7189/jogh.15.04078

**Published:** 2025-02-21

**Authors:** Junaid Iqbal, Zahra Hasan, Muhammad Atif Habib, Asma Abdul Malik, Sajid Muhammad, Kehkashan Begum, Rabia Zuberi, Muhammad Umer, Aamer Ikram, Sajid Bashir Soofi, Simon Cousens, Zulfiqar A Bhutta

**Affiliations:** 1 Department of Pediatrics and Child Health, Aga Khan University, Pakistan; 2 Department of Pathology and Laboratory Medicine, Aga Khan University, Pakistan; 3 Center of Excellence for Women and Children, Aga Khan University, Pakistan; 4 National Institute of Health, Islamabad, Pakistan; 5 London School of Hygiene and Tropical Medicine, London, UK; 6 Hospital for Sick Children, Toronto, Canada

## Abstract

**Background:**

Understanding factors associated with protective immunity against emerging viral infections is crucial for global health. Pakistan reported its first COVID-19 case on 26 February 2020, but experienced relatively low COVID-19-related morbidity and mortality between 2020 and 2022. The underlying reasons for this remain unclear, and our research aims to shed light on this crucial issue.

**Methods:**

We conducted a serial population-based serosurvey over 16 months (rounds 1–4, July 2020 to November 2021) across households in urban (Karachi) and rural (Matiari) Sindh, sampling 1100 households and 3900 individuals. We measured antibodies in sera and tested a subset of respiratory samples for COVID-19 using polymerase chain reaction (PCR) and antigen tests, also measuring haemoglobin (Hb), C-reactive protein (CRP), vitamin D, and zinc in round 1.

**Results:**

Participants showed 23% (95% confidence interval (CI) = 21.9–24.5) antibody seroprevalence in round 1, increasing across rounds 2–4 to 29% (95% CI = 27.4–30.6), 49% (95% CI = 47.2–50.9), and 79% (95% CI = 77.4–80.8), respectively. Urban residents had 2.6 times (95% CI = 1.9–3.6) higher odds of seropositivity than rural residents. Seropositivity did not differ between genders. Individuals aged 20–49 years had 7.5 (95% CI = 4.6–12.4) times higher odds of seropositivity compared to children aged 0–4 years. Most participants had no symptoms associated with COVID-19, with no reported mortality. Vitamin D deficiency was linked to seroprevalence. COVID-19 was confirmed in 1.8% of individuals tested via RT-PCR and antigen tests.

**Conclusions:**

The data suggests a steady increase in humoral immunity in Pakistan, likely due to increased transmission and associated asymptomatic disease. Overall, this reflects the longitudinal trend of protection against severe acute respiratory syndrome coronavirus 2, leading to the relatively low morbidity and mortality observed in the population.

The severe acute respiratory syndrome coronavirus 2 (SARS-CoV-2) emerged in December 2019 and subsequently caused the COVID-19 pandemic [1,2]. SARS-CoV-2 is a highly transmissible virus [3] that primarily causes a respiratory infection varying from mild to severe disease. The World Health Organization (WHO) declared COVID-19 a public health emergency of international concern on 30 January 2020 [3], and the virus was confirmed to have reached Pakistan on 26 February 2020 [4]. Pakistan has a high infectious diseases burden with limited health care infrastructure to handle a pandemic, leading to concerns that its impact would be devastating [5]. However, until global reporting of COVID-19 cases was stopped on 10 March 2023, approximately 1.7 million cases and 31 000 deaths were reported from a population of 220 million. Pakistan thus seemingly fared better in terms of morbidity and mortality compared to other countries [6], making it important to understand possible reasons behind this phenomenon.

Global data on COVID-19 cases was primarily informed by SARS-CoV-2 diagnosed in respiratory samples through PCR testing, especially in the early period of the pandemic before antigen testing was available. They were compiled in databases such as the Johns Hopkins University Coronavirus data center and Worldometer [7]. In Pakistan, data collected at local and provincial levels were collated at the National Command and Operation Center [8]. The country experienced five COVID-19 waves between 3 April 2020 and 23 February 2022 [9], wherein the number of cases reported depended on the availability of data and PCR testing capacity. As mentioned above, during the early phase of the pandemic, such testing was limited, expensive, and primarily focussed on patients with severe COVID-19 due to resource constraints [10], or on key international conduits identified by the Federal Ministry of Health [11]. Average daily PCR testing per day increased from approximately 17 000 tests during wave 1 (2020) to 45 000 during wave 3 (2021) [9]. COVID-19 antigen tests became available in Pakistan in 2022, but access to and use thereof remained limited.

Population-level seroprevalence surveys have been used to assess the proportion of individuals infected with SARS-CoV-2 and can also identify risk factors for infection, anticipate the volume of the upcoming waves, and estimate disease burden [12]. The WHO specifically recommended serial seroprevalence surveys to monitor SARS-CoV-2 infections and guide public health strategies and interventions [13].

Prior to this study, data from COVID-19 seroprevalence studies conducted in Pakistan were mainly gathered via cross-sectional sampling at a single time point in specific populations [14–17]. There was limited data from rural regions, with one nationwide study showing differing COVID-19 antibody positivity in urban and rural regions [14].

Pakistan has a predominantly young population composed of up to 50% of individuals under 40 years of age. The country is primarily agricultural, with about 37% urban and 63% rural populations [18], making it necessary to study both groups to fully understand COVID-19 rates and disease transmission. Hence, we conducted serosurveys in both urban and rural cohorts to gain insights into antibody dynamics and SARS-CoV-2 transmission trends in the population.

## METHODS

Longitudinal sampling allows one to study antibody dynamics in the population, particularly in COVID-19 waves. In this observational longitudinal study, we focussed on the province of Sindh, comparing the population of Karachi, approximately 17.6 million [19], to the population of Matiari, about 0.85 million [20], across the sixteen months encompassing four pandemic waves between July 2020 and November 2021 (Figures S1 and S2 in the **Online Supplementary Document**). We determined the prevalence of SARS-CoV-2 antibodies in the general population through serial serosurveys conducted to associate these with population demographics and health parameters. Sampling was conducted at four points within a nested cohort in the same community, which enabled us to observe temporal changes and trends in SARS-CoV-2 antibody seropositivity, providing a good understanding of the community’s response over the 18-month study period, while also testing respiratory samples in a subset of participants by PCR and COVID-19 antigen testing. Further, during the first survey, we tested blood parameters such as haemoglobin (Hb), serum C-reactive protein (CRP), vitamin D, and zinc concentrations in the study population.

### Study design

For these serial seroprevalence surveys, we adapted the WHO 30 × 7 cluster methodology to ensure population representation. We collected data all six districts in Karachi, *i.e.* Central, East, West, South, Korangi, and Malir, and from both the Hala and Matiari sub-districts in the Matiari region. We then randomly selected seven union councils from the available list in each district, which served as primary sampling units, and subsequently randomly selected 20 households from each union council to achieve the required sample size. We enrolled individuals who were residents of the respective districts for at least six months, regardless of age, with no stated intent to migrate.

Six research teams (three in Karachi and three in Matiari) collected the data and the participants’ blood samples. Each research team comprised a phlebotomist, a data collector, and a team leader. Once selected, the same households were revisited at subsequent rounds. The research staff received training on personal hygiene procedures, the use of personal protective equipment, and safe handling of laboratory samples, as well as the appropriate disposal of any waste.

The teams collected data from each participant using a structured questionnaire through a computer-assisted personal interview approach. The questionnaire included sections on demographics, exposure assessment, underlying comorbidities, and a history of any clinical symptoms like chest pain, sore throat, and fever in the past two weeks, as well as a history of travel outside their local region in the past two weeks.

Demographic and socioeconomic data related to any COVID-19-associated symptoms and clinical history were collected at each recruitment. In addition, data associated with any recent travel history was collected during the first survey round. In contrast, information on COVID-19 vaccinations was collected during the fourth survey which followed the introduction of the in March 2021 of the Sinopharm-BBIBP-CorV vaccine in Sindh, Pakistan, first introduced for vulnerable adults and front-line workers.

### Blood sample collection

After administration of the questionnaire, trained phlebotomists collected 3 ml of whole blood (one sample in each round) from each participant through venipuncture. Antibodies to SARS-CoV-2 were measured in the sera in each sample. For every individual recruited to the study, once in round 1, blood samples were tested for health-related parameters, including Hb, CRP, vitamin D, and zinc levels. The blood samples were transported to the Nutrition Research Laboratory at Aga Khan University, Karachi, under a cold chain. All samples were used for the detection of SARS-CoV-2 antibodies.

### Measurement of anti-SARS-CoV-2 antibodies and blood parameters

Antibodies against SARS-CoV-2 were measured in sera through Roche Cobas e411 automated analyzer using Roche Elecsys Anti-SARS-CoV-2 assay kit (Roche, Basel, Switzerland), per manufacturer’s instructions. The assay qualitatively detects total polyclonal antibodies (IgA, IgG, and IgM) against SARS-CoV-2 nucleocapsid (N) antigen. Before each batch, the assay was validated using positive and negative quality controls and values above the cutoff of 1.0 were considered reactive for antibodies against SARS-CoV-2. Quantitative serum 25-OH vitamin D was determined using the LIASION 25 OH Vitamin D TOTAL assay (DiaSorin, Saluggia, Italy). Zinc levels in plasma were measured using a Thermo scientific atomic absorption spectrometer. Hb levels were measured using the HemoCue Hb 301 analyzer (HemoCue, CA, USA), while CRP levels were quantified using a Roche CRP (CRPLX) kit on the Cobas c311 analyzer (Roche, Basel, Switzerland).

### Detection of SARS-CoV-2 RNA using RT-PCR

Nasopharyngeal swabs were collected by inserting a sterile swab (Virus RNA collection kit, Beaver, China) into a single nostril and rotated three to four times against the nasopharyngeal surface. Respiratory specimens were stored at −80°C until shipped to the National SARS-CoV-2 Reference Laboratory of the National Institute, Islamabad (NIH-Islamabad) on dry ice. RNA was extracted from 200μl of the media using MagMax Viral/Pathogen II Nucleic Acid Isolation Kit (Thermo Fisher Scientific Inc., Waltham, MA, USA). Reverse-transcription PCR was performed using TaqPath COVID-19 CE-IVD RT-PCR Kit on Applied Biosystems 7500 Fast Dx Real-Time PCR Instrument (Thermo Fisher Scientific Inc., Waltham, MA, USA).

### COVID-19 antigen testing of respiratory samples

Antigen testing was performed using the SARS-CoV-2 Rapid Antigen Test kit (Roche, Basel, Switzerland). Briefly, the nasopharyngeal swab samples were processed with extraction buffer vials and loaded onto the test-cartridge, with results read after 15 minutes, as per manufacturer’s instructions. Quality control for each batch run was ensured by testing the commercially available Roche SARS-CoV-2 Antigen Control kit (Roche, Basel, Switzerland).

### Sample size estimation and statistical analysis

We estimated that at least 2974 individuals (at least three individuals per household) stratified by age were needed to estimate an expected seroprevalence of 36% with 5% precision at a 95% level of confidence, with 80% power and a non-response rate of 20%. The formula we used for samples size calculation was as follows:



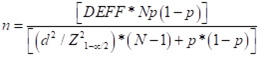



Reference calculations were based on serological data available at the time [21].

We summarised categorical variables as frequencies and proportions, and reported on seroprevalence by age, gender, socioeconomic status, clinical symptoms, type of residence (rural/urban), and district for baseline and each follow-up round. We used children aged 0–4 years as a reference group in our analyses, as they had been observed to have a lower risk of severe COVID-19 infection compared to adults [22] and as we were interested in understanding the association between seropositivity and asymptomatic infections. We determined the participants’ socioeconomic status using a wealth index derived through principal component analyses of household assets.

We used Pearson χ^2^ test to establish an association between categorical variables and trend *P*-values to compare seroprevalence over time by baseline characteristics. We explored the impact of time on the seropositive status of the respondent through mixed-effects logistic regression, whereby we incorporated individuals as a random effect to model individual heterogeneity within seropositive status. We then tested the time interaction to measure the impact of change in seropositive status. The analysis was adjusted for confounding variables like age, gender, socioeconomic status, type of residence, district, area, medical symptoms, travel history, vitamin D and zinc concentrations, and vaccination status, while retaining a positive COVID-19 antibody test as the outcome. We reported our findings using adjusted odds ratios (aOR) with 95% confidence intervals (CI). We used a Cox proportional regression model to estimate adjusted hazard ratios (aHR) and the corresponding 95% CIs for positive cases for all possible risk factors (including area, age, sex, mother tongue, household size, *etc.*).

We conducted all analyses in Stata, version 17 (Stata Corp LLC, College Station, Texas, USA), and considered a *P-*value <0.05 statistically significant.

## RESULTS

### Description of serosurveys

**Figure 1:**
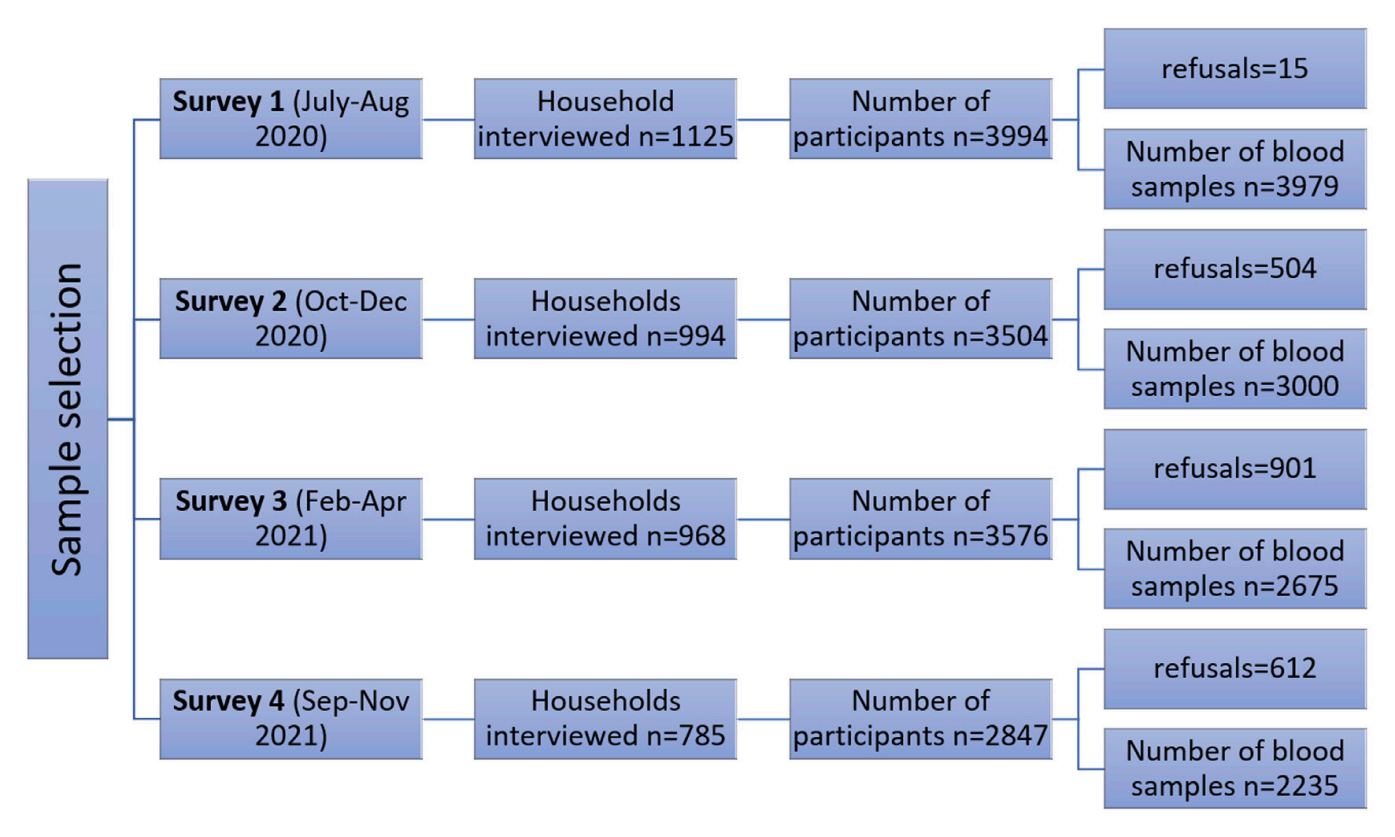
Flow diagram of sampling method and timeline of the study. The chart describes the periods of survey rounds 1–4, which were conducted between July 2020 and November 2021, as well as the number of households interviewed and the number of participants who consented to participate. The final number of individuals who participated in each round of the study are indicated.

We collected 11 889 blood samples in the four successive household serosurveys conducted through 16 months between July 2020 and November 2021 (**Figure 1**). The first survey of 1125 households was conducted during July and August 2020, with 3979 subjects consenting to interviews and submitting blood samples. The second survey was conducted from October until December 2020, with 3000 individuals from 994 households being interviewed. The third survey was conducted between February and April 2021 and included 968 households, from whom 2675 individuals were recruited. The final survey was conducted between September and November 2021, comprising 2235 participants across 785 households. In all, there were 2078 refusals to participate, comprising 0.4%, 14.4%, 25.4%, and 22.7% of successive survey rounds. In each round, a subset of individuals was selected for respiratory sampling. Nasal swabs were tested for SARS-CoV-2 RNA by PCR in rounds 1 (n = 606), 2 (n = 742), 3 (n = 316), and 4 (n = 383), and COVID-19 antigen testing in rounds 2 (n = 718), 3 (n = 315) and 4 (n = 382).

### Demographic and socioeconomic description of study subjects

The study subjects included 43.6% rural and 56.4% urban residents, with 60.4% being females (**Table 1**). Sampling was conducted in equivalent age-segregated bands across the four rounds of the serosurvey. Most individuals were classified within the ‘poor’ and ‘poorest’ (39.2%) categories. Around half (52.4%) were of Sindhi ethnicity, likely due to primary sampling within the rural area. Most participants resided in households with six or more individuals (81.7%). By round 4, COVID-19 vaccinations had been introduced across the country, so about 50% of participants in our sample reported being vaccinated. Very few individuals (n = 113, 3%) reported traveling outside the country in the previous two weeks, and even fewer (n = 71, 2%) had visited a health facility in the last two weeks. All data collected were interpreted in the context of antibody seroprevalence.

**Table 1:** Description of study subjects according to sociodemographic characteristics, presented as n (%)

	Overall, n = 11 889	Round 1, July to August 2020, n = 3979	Round 2, October to December 2020, n = 3000	Round 3, February to April 2021, n = 2675	Round 4, September to November 2021, n = 2235	*P*-value
**Location**						<0.001
Rural	5187 (43.6)	1550 (39.0)	1340 (44.7)	1176 (44.0)	1121 (50.2)	
Urban	6702 (56.4)	2429 (61.0)	1660 (55.3)	1499 (56.0)	1114 (49.8)	
**Gender**						0.200
Male	4711 (39.6)	1629 (40.9)	1158 (38.6)	1048 (39.2)	876 (39.2)	
Female	7178 (60.4)	2350 (59.1)	1842 (61.4)	1627 (60.8)	1359 (60.8)	
**Age in years**						0.930
0–4	595 (5.0)	224 (5.6)	136 (4.5)	131 (4.9)	104 (4.7)	
5–9	1171 (9.8)	401 (10.1)	283 (9.4)	273 (10.2)	214 (9.6)	
10–19	2876 (24.2)	932 (23.4)	729 (24.3)	657 (24.6)	558 (25.0)	
20–29	2159 (18.2)	739 (18.6)	562 (18.7)	463 (17.3)	395 (17.7)	
30–39	1899 (16.0)	619 (15.6)	472 (15.7)	437 (16.3)	371 (16.6)	
40–49	1474 (12.4)	481 (12.1)	384 (12.8)	334 (12.5)	275 (12.3)	
50–59	876 (7.4)	298 (7.5)	224 (7.5)	184 (6.9)	170 (7.6)	
60–69	539 (4.5)	179 (4.5)	132 (4.4)	126 (4.7)	102 (4.6)	
70–79	245 (2.1)	84 (2.1)	64 (2.1)	58 (2.2)	39 (1.7)	
80 and above	55 (0.5)	22 (0.6)	14 (0.5)	12 (0.4)	7 (0.3)	
**Wealth index quintile**						0.053
Poorest	2179 (18.3)	756 (19.0)	545 (18.2)	452 (16.9)	426 (19.1)	
Poor	2468 (20.8)	830 (20.9)	645 (21.5)	559 (20.9)	434 (19.4)	
Middle	2230 (18.8)	771 (19.4)	567 (18.9)	469 (17.5)	423 (18.9)	
Rich	2313 (19.5)	761 (19.1)	595 (19.8)	534 (20.0)	423 (18.9)	
Richest	2699 (22.7)	861 (21.6)	648 (21.6)	661 (24.7)	529 (23.7)	
**Vaccinated against COVID-19**					1113 (49.8)	<0.001

### Antibody seroprevalence in rural and urban populations

**Figure 2:**
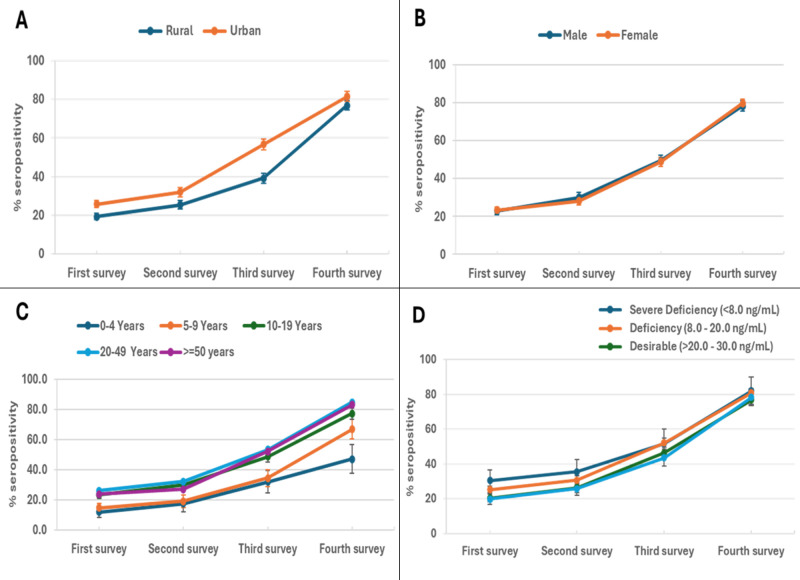
Time trends in seropositivity by urban and rural population, gender, age and vitamin D status across four survey rounds. The line graphs provide a visual representation of time trends in seropositivity (y-axis) according to different demographic and health-related subgroups throughout the four rounds of the surveys conducted. The figures show the percentage of individuals who were seropositive for antibodies measured against SARS-CoV-2 across the four different survey rounds. Data are shown as means, with standard deviations presented in error bars. **Panel A.** Comparison of urban and rural populations. **Panel B.** Comparison of male and female participants. **Panel C.** Age-wise differences between study participants (0–4, 5–9, 10–19, 20–49, and ≥50 years). **Panel D.** Seropositivity in individuals stratified according to their serum vitamin D status (severely deficient, deficient, desirable and sufficient).

We observed higher seropositivity in urban than in rural areas across the four survey rounds. COVID-19 antibody testing in study subjects showed that, during round 1 (July to August 2020), there was an overall seroprevalence of 23%, with 25.7% positivity in urban areas compared to 19.3% in the rural areas (**Figure 2**, Panel A; Table S1 in the **Online Supplementary Document**). Overall seroprevalence increased to 29% by round 2, with 31.9% positivity in urban areas *vs.* 25.4% in rural areas, with a further increase to 49% round 3 and higher (56.8%) seropositivity in urban areas compared to rural areas (39.2%). By round 4, overall seroprevalence was 79%, with 81.5% positivity in urban areas compared to 76.8% in rural areas.

### Factors associated with SARS-CoV-2 seropositivity

Seropositivity increased in all individuals throughout the four survey rounds and was similar in males and females throughout the study (**Figure 2**, Panel B, **Table 2**). Examined in age bands of 0–4, 5–9, 10–19, 20–49, and ≥50 years, it was apparent that seropositivity increased in all age groups across the four serosurveys (**Figure 2**, Panel C). We observed children aged less than 10 (groups 0–4 and 5–9 years) to have reduced seroprevalence than older age groups (Table S1 in the **Online Supplementary Document**). Seropositivity associated with vitamin D levels increased in all groups across the four serosurveys (**Figure 2**, Panel D). However, those with severe vitamin D deficiency had higher COVID-19 antibody seropositivity.

**Table 2:** Factors associated with COVID-19 antibody seroprevalence amongst study participants

	aOR (95% CI)	*P*-value*
**Round**		
1	ref	
2	1.8 (1.5–2.1)	<0.001
3	10 (8.3–11.9)	<0.001
4	128.6 (99–167)	<0.001
**Area**		
Rural	ref	
Urban	2.6 (1.9–3.6)	<0.001
**Age in years**		
0–4	ref	
5–9	1.8 (1.0–3.1)	0.052
10–19	5.1 (3.1–8.6)	<0.001
20–49	7.5 (4.6–12.4)	<0.001
≥50	6.4 (3.7–10.9)	<0.001
**Wealth index quintile**		
Poorest	0.7 (0.5–0.9)	0.018
Poor	0.9 (0.7–1.2)	0.525
Middle	1.2 (0.9–1.6)	0.258
Rich	1.3 (1.0–1.8)	0.092
Richest	ref	
**Conjunctivitis**		
Yes	1.6 (1.1–2.2)	0.007
No	ref	
**Contact with a confirmed COVID-19 case in last two weeks**		
Yes	3.2 (1.5–6.8)	0.003
No	ref	
**Vitamin D Level (μmol/L)**		
Severe Deficiency (<8.0 ng/mL)	2.1 (1.3–3.5)	0.003
Deficiency (8.0–20.0 ng/mL)	1.7 (1.3–2.3)	0.001
Desirable (>20.0–30.0 ng/mL)	1.2 (0.9–1.7)	0.211
Sufficient (>30.0 ng/mL)	ref	

aOR – adjusted odds ratio, CI – confidence interval, ref – reference

*P ≤ 0.05 considered statistically significant between groups.

Seropositivity was lower in rural (Matiari) districts than in urban Karachi districts presented in Table S1 in the **Online Supplementary Document**. Seroprevalence across the districts did not show any substantial variation throughout all rounds of surveys. Individuals living alone were less likely to be antibody-positive than individuals living in households of two or more. We also observed lower antibody seroprevalence in the poorest compared to better-off households (Table S1 in the **Online Supplementary Document**). By round 4, higher seropositivity was associated with vaccinated (86.7%) compared with unvaccinated (71.7%) individuals.

Further investigation showed that certain symptoms, such as fever or loss of smell, were only associated with COVID-19 antibody seropositivity in the univariable model. For example, vitamin D deficiency was associated with higher COVID-19 antibody seropositivity, while variations in Hb levels, CRP, or zinc levels were not associated with COVID-19 seropositivity (Table S2 in the **Online Supplementary Document**). Neither outside travel nor visiting a health facility were strongly associated with seropositivity.

In a multivariable analysis (**Table 2**), the odds of being seropositive increased with each round of the serosurvey. Further, these were 2.6 (95% CI = 1.9–3.6) times higher among urban residents than rural residents. Individuals aged 5–9, 10–19, 20–49, and ≥50 years had 1.8 (95% CI = 1.0–3.1), 5.1 (95% CI = 3.1–8.6), 7.5 (95% CI = 4.6–12.4), and 6.4 (95% CI = 3.7–10.9) times the risk of being seropositive, respectively, compared to those aged 0–4 years, after adjusting for other variables.

Having conjunctivitis was associated with a 1.6 (95% CI = 1.1–2.2) times increased risk of COVID-19 seropositivity. Reported contact with a COVID-19-positive individual in the last two weeks was also associated with higher odds of seropositivity (OR = 3.2; 95% CI = 1.5–6.8). The risk of being seropositive was 2.1 (95% CI = 1.3–3.5) times higher among those with severe vitamin D deficiency compared to those with sufficient vitamin D levels, with an additional risk of 1.7 observed in those with deficient vitamin D levels. Contact with a confirmed case of COVID-19 in the previous two weeks was associated with higher seropositive rates in the study subjects.

### Assessing risk of COVID-19 seroprevalence

We used the Cox proportional hazard model to assess time to seroconversion among study subjects, of which 79% were seropositive by round 4 (September to November 2021). Individuals aged 5–9, 10–19, 20–49, and ≥50 years had aHRs of 1.3 (95% CI = 1.0–1.7), 1.9 (95% CI = 1.5–2.5), 2.2 (95% CI = 1.7–2.7), and 2.2 (95% CI = 1.7–2.8), respectively, indicating a higher risk of seroconversion compared to those aged 0–4 years. There was no relationship between household size and risk of seroprevalence. Further, all wealth quintiles were significantly at higher risk of seroconversion but when compared against the richest quintile, the poorest had a slightly reduced risk of 0.9 (95% CI = 0.8–1.0) of seroconversion. Severe vitamin D deficiency was associated with an aHR of 1.4 (95% CI = 1.1–1.7), while vitamin D deficiency was associated with an aHR of 1.2 (95% CI = 1.1–1.4) for seropositivity (Table S3 in the **Online Supplementary Document**).

### Investigation of COVID-19 in study subjects

Antibody positivity to SARS-CoV-2 may be due to natural infection, vaccinations, or cross-reactivity to other coronaviruses [23]. To determine how the seroprevalence observed may be associated with sub-clinical infections, nasopharyngeal swab samples were collected from a subset of participants to test using COVID-19 PCR and antigen tests in 3462 samples in total across four surveys (**Table 3**). A total of 2047 PCR tests were conducted: 606 in round 1, 742 in round 2, 316 in round 3, and 383 in round 4. Of these, 37 (1.8%) were found to be COVID-19 positive. Further, 1415 respiratory samples collected between rounds 2 and 4 were tested using the COVID-19 rapid antigen assay. Of these, 26 (1.8%) were identified positive for SARS-CoV-2 antigens.

**Table 3:** Summary of COVID-19 confirmed through PCR or antigen testing of respiratory specimens of individuals tested in four serosurveys conducted from July 2020 to November 2021, presented as n (%)

Survey round	Number of tested respiratory swabs tested (PCR + antigen)	COVID-19 positive specimens	PCR tests, n	PCR positive specimens	Numberof COVID-19 antigen tests	Antigen positive specimens
Round 1	606	10 (1.7)	606	10 (1.7)		
Round 2	1460	50 (3.4)	742	26 (3.5)	718	24 (3.3)
Round 3	631	1 (0.2)	316	0 (0.0)	315	1 (0.3)
Round 4	765	2 (0.3)	383	1 (0.3)	382	1 (0.3)
Total	3462	63 (1.8)	2047	37 (1.8)	1415	26 (1.8)

PCR – polymerase chain reaction

We conducted a sensitivity analysis for seropositivity investigated through a subgroup analysis examining the relationship between COVID-19 positivity with survey rounds, using all data categories. Through multivariable analysis, we observed that COVID-19 positivity increased with survey rounds, was higher in urban areas, was higher in those aged ≥10 years, and was reduced in poorer quintiles (Table S4 in the **Online Supplementary Document**). These were all common to the trends observed in the overall study cohort. Additionally, COVID-19 positivity was associated with shortness of breath and contact with a confirmed case, while it was not associated with vitamin D deficiency.

## DISCUSSION

Based on four repeated cross-sectional surveys between July 2020 and November 2021, SARS-CoV-2 seroprevalence in two areas of Sindh showed a steady increase in the prevalence of individuals with COVID-19 antibodies over the first year of the pandemic. Specifically, we observed a constant rise in seropositivity over time in all the study districts, increasing from 23% in July 2020 to 79% by November 2021. Importantly, there was no significant increase in either reported clinical signs and symptoms or COVID-19 cases. We observed differences in seropositivity between rural areas (from 19% to 77%) and urban areas (from 26% to 82%) across the 16-month study period. We found no difference in seropositivity associated with gender, although younger age (<5 years) was associated with lower COVID-19 seropositivity. Increased household sizes and a higher wealth index were associated with higher seroprevalence rates, as was having deficient serum vitamin D levels. Notably, only 1.8% of respiratory samples tested from a subset of participants tested by RT-PCR or COVID-19 antigen testing showed the presence of SARS-CoV-2, indicating asymptomatic or sub-clinical infection in these individuals.

Of the four COVID-19 waves in Pakistan during the study period, the first occurred in May 2020, shortly before the survey began in July 2020. Three subsequent peaks followed this in December 2020, April 2021, and August 2021 [9,24]. The results of COVID-19 seroprevalence we observed during the first survey were broadly consistent with other studies conducted around the same period. A large seroprevalence study conducted in July 2020 in metropolitan cities of Pakistan reported 17.5% seropositivity [15], while another study conducted by the National Institute of Blood Diseases (NIBD) in Karachi in July 2020 reported an antibody prevalence of 34% in the community [21]. A study conducted in Lahore reported a seroprevalence of 15.6% among policemen in May 2020 [25]. By December 2020, seropositivity in a healthy blood donor population in Karachi was found to be 53% [26].

We noted an increase in refusals for participation, from 0.4% in round 1 to 22.7% in round 4, possibly due to the timing of the surveys. It is likely that there was fear regarding COVID-19 early in the pandemic and, consequently, more interest in participating in a relevant research study. However, this interest waned as fear of SARS-CoV-2 infection abated in the community.

The relatively minor increase in seroprevalence seen between the survey rounds 1 and 2 (between July and November 2020) might be explained by the countrywide ‘smart’ lockdown measures and infection mitigation measures implemented by the government from 26 November 2020 [27]. From February 2021 onwards, seroprevalence increased, as seen between the rounds 3 and 4 until November, and the increases of seroprevalence from 56.8% to 81.5% in urban and from 39.2% to 76.8% seroprevalence in rural settings, respectively. This may be attributable to the easing of lockdown measures in August 2021, with people coming back to work, poor compliance with preventive measures, and the reopening of educational institutions [28].

The early phase of the COVID-19 pandemic was associated with the predominance of the wild-type Wuhan or S clade strains of SARS-CoV-2 in the population, followed by the introduction of G clade strains by the end of 2020 [29,30]. In January 2021, alpha, the first variant of concern, was introduced in Pakistan, followed by delta variants in May 2021 [31]. COVID-19 vaccinations were rolled out on 22 February 2021, initially only for health care workers, followed by extended to groups starting with the oldest age groups [32]. Therefore, both an increase in viral transmission and vaccinations would have contributed to the increased seroconversion observed between survey rounds 3 and 4. Of note, COVID-19 vaccinations were not available for children (12–18 years) until after the end of our study period. Therefore, the increasing seroprevalence (from approximately 20% to 70%) in the 0–19-year age group was unlikely due to COVID-19 vaccinations.

We observed higher odds of seropositivity among urban dwellers, which is consistent with various regional and international studies [33–35]. The trend also fits with increased seropositivity associated with increased household size, likely pointing to higher transmission levels, such as in Karachi, which has a population of 17.6 million.

Our observation of higher odds of seropositivity among people 20–49 years old is consistent with age-related seroprevalence patterns in some studies [16,36]. This contrasts with other research that found advanced age of 60–80 years to be one of the predictors of high seroprevalence [37,38]. Such differences may be due to unique population dynamics and group mobility. Sindhi ethnicity was predominant in the study samples, as our rural cohort was from Sindh province. The identification of individuals in the population with multiple mother tongues is likely reflective of the demographic nature of Karachi, a multi-ethnic city.

Contrary to other studies, belonging to a higher wealth quintile was associated with seropositivity in our research. This contrasts with a South African study that showed a higher risk of detectable antibodies among people living in informal household settings with low socioeconomic status [39], and a population-based study in Peru that reported higher seroprevalence in socioeconomically deprived strata [40]. Differences observed in our population may be associated with the increased mobility of higher-income populations, which increases their risk of exposure to circulating SARS-CoV-2 strains.

We did not find any association between zinc micronutrients and seroconversion. However, we found vitamin D deficiency to be associated with seropositivity. Of note, a majority of individuals in Pakistan were found to have deficient vitamin D levels [41]. Vitamin D plays a key role in regulating the immune activation of cells in response to infection and may protect against SARS-CoV-2 [42]. A recent study in Pakistan reported that vitamin D-deficient individuals who tested positive for COVID-19 experienced more extended hospital stays with slower recovery and poor outcomes as compared with those who had sufficient levels [43]. However, as we had limited information regarding PCR positivity of individuals, we were unable to check for any associations between COVID-19 infections, vitamin D levels, and illness in our population. Here, vitamin D deficiency likely serves as a marker of underlying lifestyle and contextual factors such as limited sun exposure, poor dietary quality, and socioeconomic challenges [44–46] rather than acting as a direct causal factor. Therefore, there is a need for public health strategies that focus on improving nutrition and other key aspects of health which can help boost immunity and lower the risk of SARS-CoV-2 infection, especially in vulnerable groups.

Testing respiratory samples from study subjects across the four survey rounds showed that 1.8% of individuals had COVID-19. We used both PCR and antigen tests and these have different levels of sensitivity of detection for SARS-CoV-2 [47]. It is possible that some asymptomatic infections may have been missed by antigen testing, but the trend in infection rates should be consistent with our findings especially, as antibody testing was also conducted in the same cohort of individuals. We further performed a sensitivity analysis for the subgroup of individuals in whom respiratory samples were tested by COVID-19 PCR and antigen assays. We found that the seropositivity trends were the same as found in the subgroup analysis, with seropositivity associated with urban location, age greater than 10 years, and lower in the poorest population. It was also associated with shortness of breath and contact with a COVID-19 confirmed case. These data this support the overall findings of our study.

In all, our results describe increasing seroprevalence in both urban and rural regions of Sindh, Pakistan, which are most likely associated with COVID-19 immunity. These data align with reports from our center that have identified increasing IgG antibodies to SARS-CoV-2 Spike in healthy uninfected controls during the early pandemic period, likely associated with pre-pandemic immunity in the cohort [48]. The expansion of antibody responses observed here is likely driven by memory B cells triggered by natural infection and cross-reactivity to circulating alpha and beta coronaviruses [23].

The COVID-19 antibodies measured here targeted the SARS-CoV-2 nucleocapsid protein. While most information available focusses on antibodies to the spike glycoprotein, which is thought to be primarily associated with protection [49]. Antibodies reactive to the nucleocapsid protein also can have neutralising activity against SARS-CoV-2 [50]. Further, multi-parameter analysis of antibody subtypes to both spike and nucleocapsid proteins can inform on protective immunity based on both cross-reactive and SARS-CoV-2 driven immunity [51,52].

This was the first large-scale population survey that followed households over 16 months from the early pandemic period of July 2020 through four survey rounds. Its representation of baseline and subsequent seropositivity across age groups enabled us to understand population trends and the significance of the relatively low COVID-19 morbidity and mortality observed. One of the limitations of this study, however, is that some individuals refused to participate across rounds, which prevented us from testing them longitudinally. Another limitation was that we used self-reported data regarding COVID-19 related symptoms. Therefore, it is possible that participants may not have recognised mild symptoms, while others could mistakenly attribute non-COVID symptoms to the virus. Another consideration may be due to the poor socioeconomic conditions and low literacy levels of the cohort, which reduced their ability to recognise and report illness in a timely manner. Due to resource limitations, we did not confirm COVID-19 cases through PCR testing, as it was unavailable at the time. We were also unable to conduct virus-neutralising assays against SARS-CoV-2 or other related coronaviruses. Given that it is not possible to distinguish between antibodies responses to natural infection and those induced by COVID-19 vaccinations, the seropositive results of the final survey in November 2021 were attributable to both factors.

Finally, convenience sampling and enrollment from only two areas of Sindh limited the generalisability of the study to the national level.

## CONCLUSIONS

Our study presents evidence of a population-wide increase in immunity against SARS-CoV-2 infections before the introduction of COVID-19 vaccination as evidenced by rising seroprevalence in urban Karachi and rural Matiari between July 2020 and November 2021. The rising population seroprevalence coincided with COVID-19 waves in the country, but limited disease severity supports protective immunity following sub-clinical spread of the virus. This provides insights into the kinetics of protective immunity against SARS-CoV-2 during the pandemic. Such immunological data can guide further vaccine interventions as may be required by the population.

## Additional material


Online Supplementary Document
