# Supplementary material for: Evidence of rapid rise in population immunity from SARS-CoV-2 subclinical infections through pre-vaccination serial serosurveys in Pakistan
Source: J Glob Health. 2025 Feb 21;15:04078. doi: 10.7189/jogh.15.04078 (PMC11842006; doi:10.7189/jogh.15.04078)

Supplement to: Iqbal J, Hasan Z, Habib MA, Malik AA, Muhammad S, Begum K, Zuberi R, Umer M,Ikram A, Soofi SB, Cousens S, Bhutta ZA. Evidence of rapid rise in population immunity fromSARS-CoV-2 subclinical infections through pre-vaccination serial serosurveys in Pakistan. JGlob Health. 2025;15:04078.

Table S1. Seroprevalence according to sociodemographic characteristics

	First survey			Second survey			Third survey			Fourth survey		
	Positive tests	estimate [95% CI]	P-values	Positive tests	estimate [95% CI]	P-values	Positive tests	estimate [95% CI]	P-values	Positive tests	estimate [95% CI]	P-values
**Location**												
Rural	299 /1550	19.3% (17.4 - 21.3)	<0.001	340 /1340	25.4% (23.1 - 27.8)	<0.001	461 /1176	39.2% (36.4 - 42.0)	<0.001	861 /1121	76.8% (74.2 - 79.2)	0.006
Urban	624 /2429	25.7% (24.0 - 27.5)	<0.001	529 /1660	31.9% (29.7 - 34.2)	<0.001	851 /1499	56.8% (54.2 - 59.3)	<0.001	908 /1114	81.5% (79.1 - 83.7)	0.006
**Gender**												
Male	375 /1629	23.0% (21.0 - 25.1)	0.826	349 /1158	30.1% (27.6 - 32.8)	0.262	518 /1048	49.4% (46.4 - 52.5)	0.752	686 /876	78.3% (75.5 - 80.9)	0.433
Female	548 /2350	23.3% (21.7 - 25.1)	0.826	520 /1842	28.2% (26.2 - 30.3)	0.262	794 /1627	48.8% (46.4 - 51.2)	0.752	1083 /1359	79.7% (77.5 - 81.7)	0.433
**Age (Years)**												
0–4	27 /224	12.1% (8.4 - 17.0)	<0.001	24 /136	17.6% (12.1 - 25.0)	0.003	42 /131	32.1% (24.6 - 40.5)	<0.001	49 /104	47.1% (37.7 - 56.7)	0.000
5-9	59 /401	14.7% (11.6 - 18.5)	<0.001	54 /283	19.1% (14.9 - 24.1)	<0.001	94 /273	34.4% (29.0 - 40.3)	<0.001	143 /214	66.8% (60.2 - 72.8)	0.000
10-19	218 /932	23.4% (20.8 - 26.2)	0.873	217 /729	29.8% (26.6 - 33.2)	0.584	320 /657	48.7% (44.9 - 52.5)	0.841	431 /558	77.2% (73.6 - 80.5)	0.200
20-29	174 /739	23.5% (20.6 - 26.7)	0.804	169 /562	30.1% (26.4 - 34.0)	0.522	233 /463	50.3% (45.8 - 54.9)	0.546	322 /395	81.5% (77.4 - 85.0)	0.202
30-39	171 /619	27.6% (24.2 - 31.3)	0.005	148 /472	31.4% (27.3 - 35.7)	0.213	237 /437	54.2% (49.5 - 58.9)	0.018	318 /371	85.7% (81.8 - 88.9)	0.001
40-49	135 /481	28.1% (24.2 - 32.3)	0.007	140 /384	36.5% (31.8 - 41.4)	0.001	188 /334	56.3% (50.9 - 61.5)	0.005	242 /275	88.0% (83.6 - 91.3)	0.000
50-59	73 /298	24.5% (19.9 - 29.7)	0.581	58 /224	25.9% (20.6 - 32.0)	0.292	96 /184	52.2% (45.0 - 59.3)	0.380	149 /170	87.6% (81.8 - 91.8)	0.005
60-69	45 /179	25.1% (19.3 - 32.0)	0.529	40 /132	30.3% (23.1 - 38.7)	0.729	67 /126	53.2% (44.4 - 61.7)	0.343	77 /102	75.5% (66.2 - 82.9)	0.353
70-79	17 /84	20.2% (13.0 - 30.2)	0.517	16 /64	25.0% (15.9 - 37.0)	0.480	31 /58	53.4% (40.7 - 65.8)	0.498	32 /39	82.1% (66.9 - 91.2)	0.653
80 and above	4 /22	18.2% (7.0 - 39.6)	0.578	3 /14	21.4% (7.1 - 49.4)	0.536	4 /12	33.3% (13.1 - 62.4)	0.284	6 /7	85.7% (41.9 - 98.0)	0.672
**District**												
Matiari	299 /1550	19.3% (17.4 - 21.3)	<0.001	340 /1340	25.4% (23.1 - 27.8)	<0.001	461 /1176	39.2% (36.4 - 42.0)	<0.001	861 /1121	76.8% (74.2 - 79.2)	0.006
Karachi Central	113 /498	22.7% (19.2 - 26.6)	0.775	94 /345	27.2% (22.8 - 32.2)	0.454	196 /306	64.1% (58.5 - 69.2)	<0.001	236 /281	84.0% (79.2 - 87.8)	0.034
Karachi East	133 /529	25.1% (21.6 - 29.0)	0.255	92 /324	28.4% (23.7 - 33.6)	0.810	152 /280	54.3% (48.4 - 60.0)	0.064	175 /220	79.5% (73.7 - 84.4)	0.879
Karachi South	85 /323	26.3% (21.8 - 31.4)	0.166	62 /244	25.4% (20.3 - 31.2)	0.202	116 /221	52.5% (45.9 - 59.0)	0.286	89 /124	71.8% (63.2 - 79.0)	0.039
Karachi West	182 /587	31.0% (27.4 - 34.9)	<0.001	179 /455	39.3% (35.0 - 43.9)	<0.001	224 /401	55.9% (51.0 - 60.7)	0.003	275 /325	84.6% (80.3 - 88.1)	0.009
Korangi	57 /212	26.9% (21.3 - 33.3)	0.192	59 /147	40.1% (32.5 - 48.3)	0.002	79 /143	55.2% (47.0 - 63.2)	0.128	82 /95	86.3% (77.8 - 91.9)	0.082
Malir	54 /280	19.3% (15.1 - 24.3)	0.109	43 /145	29.7% (22.8 - 37.6)	0.851	84 /148	56.8% (48.7 - 64.5)	0.054	51 /69	73.9% (62.3 - 82.9)	0.278
**HH Size**												
1	1 /10	10.0% (1.4 - 46.7)	0.342	1 /5	20.0% (2.7 - 69.1)	0.661	2 /6	33.3% (8.4 - 73.2)	0.449	4 /7	57.1% (23.0 - 85.6)	0.170

2	38 /143	26.6% (20.0 - 34.4)	0.331	38 /114	33.3% (25.3 - 42.5)	0.295	43 /93	46.2% (36.4 - 56.4)	0.581	73 /89	82.0% (72.6 - 88.7)	0.497
3	15 /81	18.5% (11.5 - 28.5)	0.315	15 /54	27.8% (17.5 - 41.1)	0.846	27 /53	50.9% (37.7 - 64.0)	0.780	30 /40	75.0% (59.4 - 86.0)	0.515
4-5	135 /542	24.9% (21.4 - 28.7)	0.310	109 /386	28.2% (24.0 - 32.9)	0.735	158 /311	50.8% (45.3 - 56.3)	0.510	200 /246	81.3% (75.9 - 85.7)	0.379
6 and above	734 /3203	22.9% (21.5 - 24.4)	0.394	706 /2441	28.9% (27.2 - 30.8)	0.911	1082 /2212	48.9% (46.8 - 51.0)	0.766	1462 /1853	78.9% (77.0 - 80.7)	0.520
**Wealth Quintile**												
Poorest	154 /756	20.4% (17.6 - 23.4)	0.041	133 /545	24.4% (21.0 - 28.2)	0.010	182 /452	40.3% (35.8 - 44.9)	<0.001	320 /426	75.1% (70.8 - 79.0)	0.023
Poor	172 /830	20.7% (18.1 - 23.6)	0.058	167 /645	25.9% (22.7 - 29.4)	0.052	251 /559	44.9% (40.8 - 49.1)	0.028	340 /434	78.3% (74.2 - 82.0)	0.644
Middle	192 /771	24.9% (22.0 - 28.1)	0.212	183 /567	32.3% (28.6 - 36.2)	0.054	247 /469	52.7% (48.1 - 57.2)	0.085	345 /423	81.6% (77.6 - 85.0)	0.176
Rich	203 /761	26.7% (23.7 - 29.9)	0.012	188 /595	31.6% (28.0 - 35.4)	0.114	291 /534	54.5% (50.2 - 58.7)	0.005	349 /423	82.5% (78.6 - 85.8)	0.060
Richest	202 /861	23.5% (20.7 - 26.4)	0.836	198 /648	30.6% (27.1 - 34.2)	0.314	341 /661	51.6% (47.8 - 55.4)	0.132	415 /529	78.4% (74.7 - 81.7)	0.650
**Mother tongue**												
Urdu	163 /716	22.8% (19.8 - 26.0)	0.763	137 /538	25.5% (22.0 - 29.3)	0.048	286 /530	54.0% (49.7 - 58.2)	0.012	333 /425	78.4% (74.2 - 82.0)	0.653
Punjabi	105 /376	27.9% (23.6 - 32.7)	0.023	83 /223	37.2% (31.1 - 43.8)	0.005	83 /146	56.8% (48.7 - 64.6)	0.053	76 /95	80.0% (70.8 - 86.9)	0.835
Sindhi	379 /1936	19.6% (17.9 - 21.4)	<0.001	432 /1596	27.1% (24.9 - 29.3)	0.015	647 /1462	44.3% (41.7 - 46.8)	<0.001	994 /1277	77.8% (75.5 - 80.0)	0.078
Pashto	87 /287	30.3% (25.3 - 35.9)	0.003	74 /206	35.9% (29.7 - 42.7)	0.023	82 /144	56.9% (48.7 - 64.8)	0.052	128 /153	83.7% (76.9 - 88.7)	0.156
Balochi	53 /166	31.9% (25.3 - 39.4)	0.007	35 /111	31.5% (23.6 - 40.7)	0.544	52 /97	53.6% (43.7 - 63.3)	0.361	66 /79	83.5% (73.7 - 90.2)	0.329
Saraiki	10 /41	24.4% (13.7 - 39.7)	0.856	7 /17	41.2% (21.0 - 64.8)	0.272	9 /24	37.5% (20.8 - 57.8)	0.260	5 /8	62.5% (28.5 - 87.5)	0.259
Hindko	10 /36	27.8% (15.6 - 44.4)	0.514	6 /18	33.3% (15.8 - 57.1)	0.683	8 /19	42.1% (22.6 - 64.4)	0.545	11 /17	64.7% (40.4 - 83.2)	0.150
Others	116 /421	27.6% (23.5 - 32.0)	0.025	95 /291	32.6% (27.5 - 38.2)	0.146	145 /253	57.3% (51.1 - 63.3)	0.006	156 /181	86.2% (80.4 - 90.5)	0.016

The number of samples with a positive as compared with a negative antibody seroprevalence result were compared between subgroups and those with p-values ≤ 0.05 were considered to significantly different

Table S2. Risk factors associated with COVID-19 antibody seropositivity

	Unadjusted OR 95% CI	P-values	Adjusted OR 95% CI	P-values
**Survey Round**				
1	Ref.		Ref.	
2	1.8 (1.5,2.0)	<0.001	1.8 (1.5,2.1)	<0.001
3	9.7 (8.2,11.6)	<0.001	10 (8.3,11.9)	<0.001
4	124.3 (95.9,161.1)	<0.001	128.6 (99,167)	<0.001
**Area**				
Rural	Ref.		Ref.	
Urban	1.3 (1.2,1.5)	<0.001	2.6 (1.9,3.6)	<0.001
**Gender**				
Male	Ref.			
Female	1.0 (0.9,1.2)	0.745		
**Age**				
0-4 Years	Ref.		Ref.	
5-9 Years	1.5 (1.1,2.2)	0.017	1.8 (1.0,3.1)	0.052
10-19 Years	2.9 (2.1,3.9)	<0.001	5.1 (3.1,8.6)	<0.001
20-49 Years	3.5 (2.6,4.7)	<0.001	7.5 (4.6,12.4)	<0.001
>=50 years	3.0 (2.2,4.2)	<0.001	6.4 (3.7,10.9)	<0.001
**Mother tongue**				
Urdu	Ref.		Ref.	
Punjabi	0.9 (0.7,1.2)	0.6	1.8 (1.2,2.7)	0.007
Sindhi	0.9 (0.7,1.0)	0.06	1.5 (1.0,2.2)	0.028
Pashto	1.3 (1.0,1.7)	0.036	2.1 (1.3,3.3)	0.001
Balochi	1.2 (0.8,1.6)	0.339	1.9 (1.1,3.3)	0.022
Saraiki	0.6 (0.3,1.2)	0.147	1.4 (0.5,4.2)	0.508

	Unadjusted OR 95% CI	*P*-values	Adjusted OR 95% CI	*P*-values
Hindko	0.9 (0.4,1.7)	0.645	0.8 (0.3,2.3)	0.615
Others	1.1 (0.9,1.4)	0.372	1.5 (1.0,2.2)	0.054
**Number of family members**				
1	Ref.			
2	2.2 (0.6,8.3)	0.231		
3	1.5 (0.4,5.9)	0.53		
4-5	1.9 (0.5,6.8)	0.329		
6 and above	1.9 (0.5,6.7)	0.332		
**Wealth Index(quintiles)**				
Poorest	0.7 (0.6,0.9)	<0.001	0.7 (0.5,0.9)	0.018
Poor	0.8 (0.6,0.9)	0.006	0.9 (0.7,1.2)	0.525
Middle	1.0 (0.8,1.2)	0.821	1.2 (0.9,1.6)	0.258
Rich	1.1 (0.9,1.3)	0.291	1.3 (1.0,1.8)	0.092
Richest	Ref.		Ref.	
**Fever**				
Yes	1.9 (1.7,2.1)	<0.001		
No	Ref.			
**Loss of Smell**				
Yes	2.8 (2,3.9)	<0.001		
No	Ref.			
**Loss of Taste**				
Yes	3.0 (2.2,4.1)	<0.001		
No	Ref.			
**Dry cough**				
Yes	1.4 (1.2,1.6)	<0.001		
No	Ref.			

	Unadjusted OR 95% CI	*P*-values	Adjusted OR 95% CI	*P*-values
**Sore throat**				
Yes	1.3 (1.1,1.5)	0.01		
No	Ref.			
**Diarrhea**				
Yes	1.6 (1.3,2)	<0.001		
No	Ref.			
**Difficulty breathing or shortness of breath/Chest pain or pressure**				
Yes	2.0 (1.6,2.5)	<0.001		
No	Ref.			
**Loss of speech or movement /Weakness/Tiredness**				
Yes	2.3 (2.0,2.6)	<0.001		
No	Ref.			
**Aches and pains/Headache**				
Yes	2.3 (2,2.6)	<0.001		
No	Ref.			
**Conjunctivitis**				
Yes	2.8 (2.2,3.6)	<0.001	1.6 (1.1,2.2)	0.007
No	Ref.		Ref.	
**Chronic disease**				
Yes	1.2 (1.0,1.4)	0.044		
No	Ref.			
**Travelled in the las 2 weeks**				
Yes	1.2 (0.9,1.7)	0.264		
No	Ref.			
**Visited any health care facility in last 2 weeks**				

	Unadjusted OR 95% CI	*P*-values	Adjusted OR 95% CI	*P*-values
Yes	1.4 (0.9,2.3)	0.125		
No	Ref.			
**Contact with a confirmed COVID case in last 2 weeks**				
Yes	6.1 (3.7,10.1)	<0.001	3.2 (1.5,6.8)	0.003
No	Ref.		Ref.	
**Hemoglobin level**				
Severe deficiency (<7 gm/dL)	1.3 (0.4,4.2)	0.612		
Moderate deficiency (7 - 11.99 gm/dL)	0.9 (0.8,1.1)	0.357		
Normal (>= 12 gm/dL)	Ref.			
**Vitamin D Level (µmol/L)**				
Severe Deficiency (<8.0 ng/mL)	1.6 (1.2,2.2)	0.002	2.1 (1.3,3.5)	0.003
Deficiency (8.0 - 20.0 ng/mL)	1.3 (1.1,1.6)	0.001	1.7 (1.3,2.3)	0.001
Desirable (>20.0 - 30.0 ng/mL)	1.1 (0.9,1.3)	0.407	1.2 (0.9,1.7)	0.211
Sufficient (>30.0 ng/mL)	Ref.		Ref.	
**C-reactive protein Level**				
Normal (<=0.5)	Ref.			
Elevated (>0.5)	1.0 (0.9,1.2)	0.81		
**Zinc**				
Deficient (<60 µg/dL)	0.9 (0.8,1.0)	0.194		
Non-Deficient (>=60 µg/dL)	Ref.			

OR – odds ratio, Ref – reference group, CI – confidence interval

Risk of positive antibodies was determined by unadjusted and adjusted ORs and respective p-values. All potential covariates (P-values <0.25 in the univariate analysis) included in the multivariable analysis and were dropped consecutively based on statistical significance. The variables having p-value <0.05 retained in the model for final adjustment.

Table S3. Cox proportional regression model for overall time to seropositivity among study participants from July 2020 to November 2021

	Unadjusted HR 95% CI	P-values	Adjusted HR 95% CI	P-values
**Area**				
Rural	Ref.			
Urban	1.0 (0.9,1.1)	0.869		
**Gender**				
Male	Ref.			
Female	1.1 (1,1.2)	0.039		
**Age**				
0-4 Years	Ref.		Ref.	
5-9 Years	1.4 (1.1,1.8)	0.017	1.3 (1.0,1.7)	0.035
10-19 Years	2.1 (1.6,2.6)	<0.001	1.9 (1.5,2.5)	<0.001
20-49 Years	2.3 (1.8,2.8)	<0.001	2.2 (1.7,2.7)	<0.001
>=50 years	2.2 (1.7,2.8)	<0.001	2.2 (1.7,2.8)	<0.001
**Number of family members**				
1	Ref.			
2	1.5 (0.7,3.5)	0.309		
3	1.5 (0.6,3.5)	0.368		
4-5	1.6 (0.7,3.6)	0.249		
6 and above	1.5 (0.7,3.4)	0.31		
**Wealth Index(quintiles)**				
Poorest	0.9 (0.8,1.0)	0.058	0.9 (0.8,1.0)	0.048
Poor	1.1 (1.0,1.2)	0.266	1.1 (0.9,1.2)	0.342
Middle	1.1 (0.9,1.2)	0.316	1.1 (0.9,1.2)	0.301
Rich	1.2 (1.0,1.3)	0.016	1.1 (1.1,1.3)	0.047
Richest	Ref.		Ref.	

**Travelled in the las 2 weeks**				
Yes	1.2 (1,1.5)	0.06		
No	Ref.			
**Visited any health care facility in last 2 weeks**				
Yes	1.1 (0.8,1.4)	0.613		
No	Ref.			
**Hemoglobin level**				
Severe deficiency (<7 gm/dL)	1.2 (0.6,2.3)	0.652		
Moderate deficiency (7 - 11.99 gm/dL)	1.0 (0.9,1.1)	0.521		
Normal (>= 12 gm/dL)	Ref.			
**Vitamin D Level (µmol/L)**				
Severe Deficiency (<8.0 ng/mL)	1.4 (1.2,1.7)	<0.001	1.4 (1.1,1.7)	0.001
Deficiency (8.0 - 20.0 ng/mL)	1.3 (1.1,1.4)	<0.001	1.2 (1.1,1.4)	<0.001
Desirable (>20.0 - 30.0 ng/mL)	1.1 (1.0,1.2)	0.174	1.1 (1.0,1.2)	0.12
Sufficient (>30.0 ng/mL)	Ref.		Ref.	
**C-reactive protein Level**				
Normal (<=0.5)	Ref.			
Elevated (>0.5)	1.1 (1,1.3)	0.03		
**Zinc**				
Deficient (<60 µg/dL)	1.0 (0.9,1.1)	0.997		
Non-Deficient (>=60 µg/dL)	Ref.			

HR – hazard ratio, Ref – reference group, CI – confidence interval

The risk of COVID-19 antibody seropositivity was determined using the Cox's proportional regression model with unadjusted and adjusted hazard ratios using to identify significant differences between sub-groups, p-values ≤ 0.05 are considered significant.

Table S4. Subgroup analysis: Risk factors associated with COVID-19 seropositivity

	Unadjusted OR 95% CI	*P*-values	Adjusted OR 95% CI	*P*-values
**Round**				
1	Ref.		Ref.	
2	1.9 (1.5,2.3)	<0.001	1.8 (1.5,2.3)	<0.001
3	10.6 (8.3,13.5)	<0.001	10.6 (8.3,13.4)	<0.001
4	157.3 (113.1,218.7)	<0.001	151.8 (109.1,211.3)	<0.001
**Area**				
Rural	Ref.		Ref.	
Urban	1.5 (1.3,1.8)	<0.001	3 (1.8,4.9)	<0.001
**Gender**				
Male	Ref.			
Female	0.9 (0.8,1.1)	0.325		
**Age**				
0-4 Years	Ref.			Ref.
5-9 Years	1.4 (0.9,2.3)	0.183	1.9 (0.8,4.7)	0.143
10-19 Years	2.7 (1.7,4.2)	<0.001	7.1 (3.2,15.8)	<0.001
20-49 Years	3.3 (2.1,5.1)	<0.001	10 (4.6,21.9)	<0.001
>=50 years	2.9 (1.8,4.6)	<0.001	7.1 (3.1,16.4)	<0.001
**Number of family members**				
1	Ref.			
2	4.3 (0.7,27.4)	0.125		
3	4.4 (0.7,29.4)	0.127		
4-5	3.8 (0.6,23.6)	0.155		
6 and above	4.3 (0.7,26.3)	0.117		
**Wealth Index(quintiles)**				
Poorest	0.6 (0.5,0.8)	0.001	0.4 (0.3,0.7)	<0.001
Poor	0.7 (0.6,0.9)	0.003	0.5 (0.3,0.8)	0.006
Middle	1.0 (0.8,1.3)	0.793	0.9 (0.6,1.5)	0.771
Rich	1.1 (0.8,1.3)	0.657	1.0 (0.6,1.5)	0.935

Richest	Ref.		Ref.	
**Fever**				
Yes	1.9 (1.7,2.2)	<0.001		
No	Ref.			
**Loss of Smell**				
Yes	3.1 (2.1,4.6)	<0.001		
No	Ref.			
**Loss of Taste**				
Yes	3.6 (2.4,5.3)	<0.001		
No	Ref.			
**Dry cough**				
Yes	1.4 (1.2,1.7)	<0.001		
No	Ref.			
**Sore throat**				
Yes	1.2 (1.0,1.5)	0.043		
No	Ref.			
**Diarrhoea**				
Yes	1.4 (1.1,1.8)	0.013		
No	Ref.			
**Difficulty breathing or shortness of breath/Chest pain or pressure**				
Yes	2.2 (1.7,2.9)	<0.001	1.8 (1.2,2.7)	0.003
No	Ref.		Ref.	
**Loss of speech or movement /Weakness/Tiredness**				
Yes	2.6 (2.2,3.0)	<0.001		
No	Ref.			
**Aches and pains/Headache**				
Yes	2.5 (2.1,2.9)	<0.001		
No	Ref.			
**Conjunctivitis**				
Yes	2.9 (2.2,4.0)	<0.001		
No	Ref.			
**Chronic disease**				

Yes	1.3 (1.0,1.6)	0.049		
No	Ref.			
**Travelled in the las 2 weeks**				
Yes	1.1 (0.7,1.8)	0.631		
No	Ref.			
**Visited any health care facility in last 2 weeks**				
Yes	1.5 (0.7,3.0)	0.311		
No	Ref.			
**Contact with a confirmed COVID case in last 2 weeks**				
Yes	7.3 (4.0,13.4)	<0.001	3.1 (1.3,7.6)	0.012
No	Ref.		Ref.	
**Hemoglobin level**				
Severe deficiency (<7 gm/dL)	2.1 (0.3,14.0)	0.447		
Moderate deficiency (7 - 11.99 gm/dL)	0.9 (0.8,1.1)	0.363		
Normal (>= 12 gm/dL)	Ref.			
**Vitamin D Level (µmol/L)**				
Severe Deficiency (<8.0 ng/mL)	1.3 (0.9,2.0)	0.151		
Deficiency (8.0 - 20.0 ng/mL)	1.2 (1.0,1.5)	0.089		
Desirable (>20.0 - 30.0 ng/mL)	0.9 (0.7,1.2)	0.536		
Sufficient (>30.0 ng/mL)	Ref.			
**CRP Level**				
Normal (<=0.5)	Ref.			
Elevated (>0.5)	0.9 (0.7,1.1)	0.429		
**Zinc**				
Deficient (<60 µg/dL)	0.9 (0.8,1.1)	0.286		
Non-Deficient (>=60 µg/dL)	Ref.			

OR – odds ratio, Ref – reference group, CI – confidence interval

Risk of positive antibodies was determined by unadjusted and adjusted ORs and respective p-values conducted in individuals in whom respiratory samples were tested for COVID-19 by PCR and antigen tests. All potential covariates (P-values <0.25 in the univariate analysis) included in the multivariable analysis and were dropped consecutively based on statistical significance. The variables having p-value <0.05 retained in the model for final adjustment.

Figure S1. COVID-19 cases in Pakistan during the study period. The figure illustrates the number of new confirmed COVID-19 cases reported in Pakistan during the study period, from July 2020 and November 2021. This data was sourced from the Johns Hopkins University COVID-19 Data Repository.

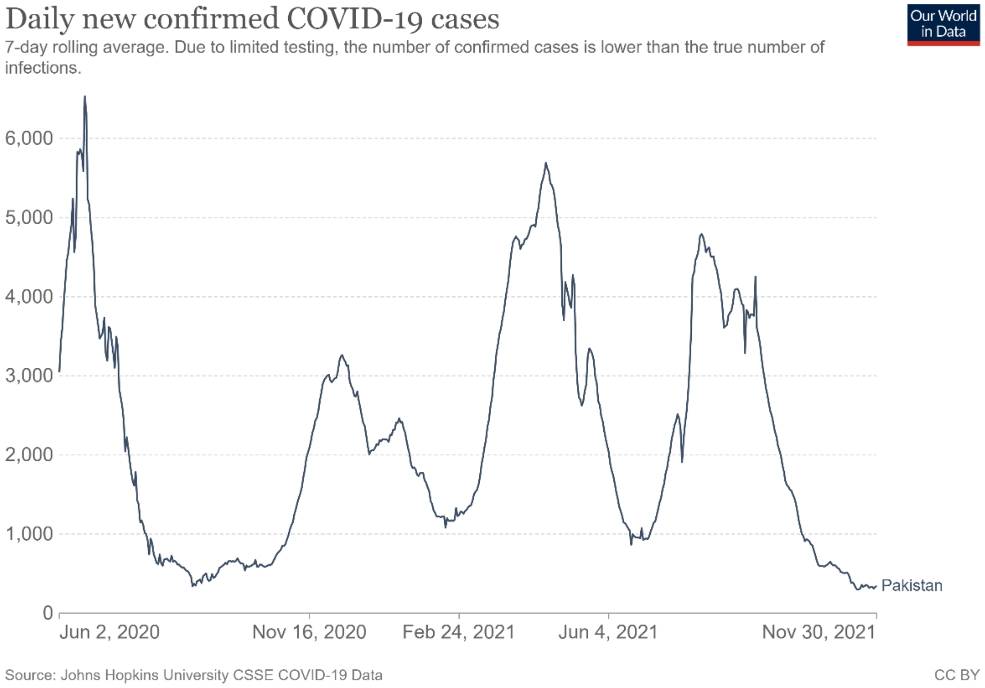

Figure S2. Map of study sites and change in antibody prevalence against SARS-CoV-2. The figure illustrates the spatial distribution and variation in seroprevalence changes across the selected study sites. The main map details Karachi city, the upper inset shows a map of Pakistan highlighting the locations of Karachi city, while the lower inset zooms in on district Matiari. The colored symbols on the map indicate the change in prevalence of **antibodies against SARS-CoV-2 between survey rounds (R4 - R1) across different areas; blue, > 60%; green, 50.1-60%; yellow, 40.1-50% and red, ≤40%, respectively.**

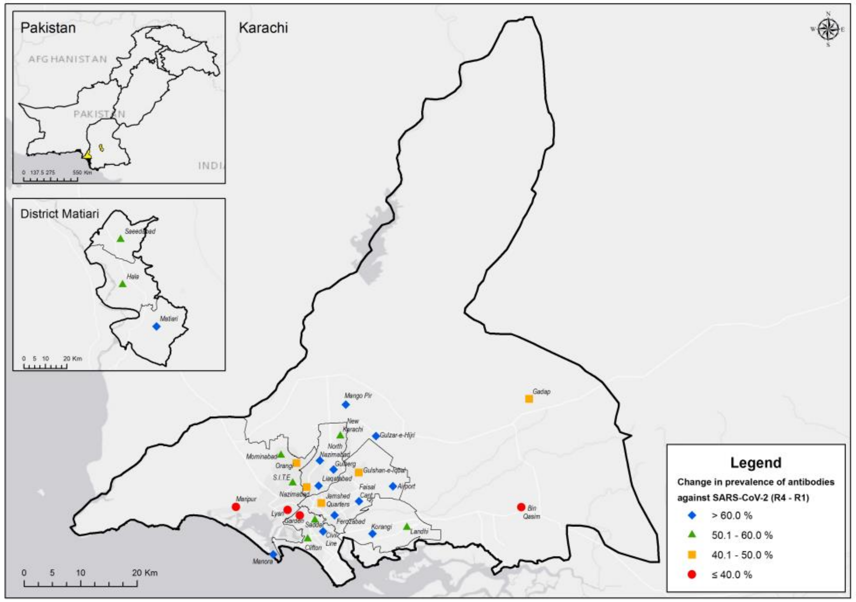

Supplement: Online Supplementary Document [file jogh-15-04078-s001.pdf]
